# Disconnective hemispherotomy: technique and operative highlights

**DOI:** 10.3171/2024.4.FOCVID2436

**Published:** 2024-07-01

**Authors:** Akshay Sharma, Richard Rammo, Nehaw Sarmey, Efstathios D. Kondylis, Demitre Serletis, William Bingaman

**Affiliations:** 1 Epilepsy Center, Neurological Institute, Cleveland Clinic, Cleveland;; 2 Department of Neurological Surgery, Neurological Institute, Cleveland Clinic, Cleveland; and; 3 Cleveland Clinic Lerner College of Medicine of Case Western Reserve University, Cleveland, Ohio

**Keywords:** epilepsy, hemispherectomy, hemispherotomy, resection, cerebral hemisphere, pediatrics

## Abstract

Hemispherectomy is an effective procedure used in the treatment of drug-resistant hemispheric epilepsy, especially in the pediatric population. A number of resective and disconnective techniques are used, and selection of surgical strategy is paramount to achieving successful results. Notably, disconnective (or functional) hemispherotomy maximizes the benefits of safe, surgical disconnection while minimizing hemispheric tissue resection, thereby avoiding some of the perioperative factors contributing to morbidity in traditional anatomical hemispherectomy procedures. In this video, the authors outline the principal surgical steps of disconnective hemispherotomy and highlight important technical factors leading to optimal outcomes in patients with refractory, oftentimes catastrophic, hemispheric epilepsy.

The video can be found here: https://stream.cadmore.media/r10.3171/2024.4.FOCVID2436

## Transcript

Hemispherectomy is a powerful tool in the treatment of drug-resistant hemispheric epilepsy. The operation has been modified by numerous surgeons^1–4^ to reduce complications and morbidity, namely hydrocephalus and superficial cerebral hemosiderosis.^5,6^ Here we will describe the functional, or disconnective, technique, which provides surgical disconnection of the hemisphere while minimizing tissue resection.

### 0:39 Technique Selection.

Technique selection should be guided by specific patient anatomy and underlying pathology. Consideration should be given to the best approach to achieve optimal visualization of the connected structures and their subsequent disconnection. All hemispherectomy approaches should be considered disconnective procedures, with varying levels of tissue sparing. As such, technique choice should be made in order to ensure optimal disconnection is completed, as this is the strongest predictor of patient outcome and seizure freedom.^7,8^

### 1:06 Operative Preparation.

The patient is placed under general endotracheal anesthesia. An arterial line, indwelling catheter, and large-bore venous access are placed by the anesthesia and nursing teams. Intravenous steroids and antibiotics are administered within an hour of skin incision. Electrocorticography, cortical stimulation, and neuromonitoring are not routinely used during surgery. Blood products should be placed on hold for the operating room, as especially in pediatric patients, operative blood loss can lead to hemodynamic instability and coagulopathies.

### 1:35 Positioning and Incision.

We position the patient supine with the head in a 90° lateral turn with a support under the ipsilateral shoulder. The vertex is placed slightly down to allow access to the mesial temporal and interhemispheric structures.

A large hemispheric incision is planned, either T-shaped, barn-door, or question mark in fashion. The incision extends ventrally to the zygoma to allow full exposure of the temporal lobe.

### 1:56 Opening.

Here we present a right-sided approach. The skin and temporalis can be reflected together or separately to expose a craniotomy site that includes the coronal suture anteriorly, the asterion posteriorly, the midconvexity in the dorsal extent, and to the floor of the middle fossa ventrally.^2^ This patient has a preexisting ventricular shunt which is preserved, disconnected, and protected. The bone flap is turned to maximize visualization, which ensures adequate disconnection and minimized tissue injury from extensive retraction. The dura is tacked to the bone to prevent the formation of postoperative epidural collections. The dura is opened in C-shaped fashion and reflected along a frontotemporal base.

### 3:09 Hemispherotomy.

Five major disconnections are needed in order to successfully complete the operation. Resection of the mesial temporal parietal lobe, disruption of the corona radiata and temporal white matter, a corpus callosotomy, disconnection of the basal frontal connections, and finally removal of the insula.

### 3:25 Opening of the Ventricle.

The sylvian fissure is identified. A pial cut is made in the dorsal superior temporal gyrus and the temporal lobe is elevated from the pia overlying the vessels within the sylvian fissure. Here the lateral temporal lobe is elevated from the insula laterally and the inferior circular sulcus is identified below. The frontal lobe is then gently retracted to expose the superior circular sulcus. This connects back to the inferior circular sulcus, which serves as a landmark for the temporal horn of the lateral ventricle. The temporal horn is opened to expose the mesial temporal structures. Cottonoids are used to prevent blood from entering the contralateral ventricular system. As the ventricle is exposed, several key anatomical landmarks are identified, including the hippocampus, amygdala, and choroidal point. The ventricle is unroofed completely to expose the rest of the hippocampal body and tail as it transitions into the fornix. Above the sylvian fissure, the corona radiata is divided to enter the lateral ventricle. Here the corpus callosum can be visualized at the junction of the septum pellucidum and the ventricular roof. Again, a small cotton ball can be used to plug the foramen of Monro. The frontal parietal white matter should be resected posteriorly and around the posterior insula to connect with the infra-sylvian dissection.

### 5:12 Basal Frontal Disconnection.

Attention is then turned to the horizontal frontal fibers which lie anterior and superior to the insula. Attention to this area is particularly important as it is frequently a site of residual connection that requires reoperation, especially when malformative cortex is present in this region. The posterior basal frontal lobe is removed to expose the orbitofrontal pia, which is then coagulated and opened down to the olfactory nerve. Here the gyrus rectus is identified and aspirated until the pia of the contralateral gyrus rectus is seen. Here the origin of the A2 branches can be visualized and followed to the genu of the corpus callosum, which generally occurs as the arteries curve posteriorly into the A3 segment. This anatomical boundary is important to note, as it allows for ample disconnection at the genu without injurious extension into the hypothalamus ventral to the genu. Dissection should be subpial with preservation of as much of the vascular supply to the cortex left behind as possible to avoid postoperative issues with stroke and brain swelling.

### 6:10 Corpus Callosotomy.

The curve of the genu can be confusing, and one must remember that two layers of gray matter and pia will be encountered during its aspiration. The callosum should be disconnected at the junction of the roof of the ventricle and the septum pellucidum. Aspiration of the ipsilateral cingulate ensures a complete disconnection and makes postoperative imaging easier to interpret. The inferior edge of the falx is often seen and serves as an excellent anatomical reference during callosotomy. Branches of the anterior cerebral artery should be spared whenever possible. The surgeon must respect both arteries above the callosum, as it is difficult to tell which artery supplies the affected hemisphere. Care should be taken to completely disconnect the splenium. Review of the individual callosal anatomy as seen on the preoperative MRI helps as the callosal anatomy can vary depending on the etiology of the epilepsy.

### 7:05 Mesial Temporal Resection and Final Splenium Removal.

Attention is then turned to the mesial temporal structures, which were previously exposed. The choroidal point is identified and the amygdala is dissected along the plane connecting the M1 segment of the MCA and the choroidal point. The remaining parahippocampus and uncus are then removed via subpial aspiration, being careful not to violate the mesial pia and/or injure the structures in the perimesencephalic cistern. The hippocampus is then reflected inferiorly and the choroidal fissure opened by aspiration of the fimbria/fornix. At this point, the hippocampal sulcus is identified and developed with further aspiration of the dentate gyrus. Once visualized, the hippocampal sulcus including the hippocampal arteries and veins are coagulated and divided. The hippocampus is then removed in one segment for pathological study.

In this case, we now remove the lateral neocortical structures, to allow further exposure of the posterior mesial temporal region. The lateral neocortex can also be removed early on during the exposure of the mesial structures at the beginning of the case, by connecting sagittal and coronal cuts through the lateral ventricular sulcus along the collateral eminence. This can be an important tool to aid in surgical visualization and help to guarantee an adequate disconnection. The tail of the hippocampus is then followed posteriorly around the midbrain into the forniceal columns where it is truncated. Above the forniceal columns, note the relation of the splenium. This anatomical relationship allows inspection and complete disconnection of the callosum.

### 9:06 Removal of the Insula.

The final step of the operation is the removal of the insula. With care to preserve the vessels overlying, the gyri are gently aspirated with suction. Excessive removal of tissue can lead to disruption of the basal ganglia deep to the insula, which is usually identified by its speckled appearance and the incidence of diffuse bleeding on aspiration.

### 9:28 Closure.

Hemostasis is achieved, with care to remove as much blood from the foramen of Monro as possible. A ventricular drain is placed into the subdural space in the ventricular cavity. The dura is then closed primarily. The shunt is reconnected. For patients with adjustable shunts, we typically turn the shunt to the highest resistance to decrease postoperative fluid flow through the system, until the ventricular drain is removed between postoperative day 3 through 5.^9^ The bone is replaced, and the muscle is reapproximated. A subgaleal drain is also placed, and the skin closed. Patients are placed on an extended steroid taper after surgery and antiseizure medications are restarted at preoperative doses.

## Acknowledgments

We acknowledge Gwendolyn Fuller, MFA, for composition of the illustrations presented in the video.

## Disclosures

The authors report no conflict of interest concerning the materials or methods used in this study or the findings specified in this publication.

## Author Contributions

Primary surgeon: Bingaman. Assistant surgeon: Sharma, Kondylis. Editing and drafting the video and abstract: Bingaman, Sharma, Sarmey, Kondylis, Serletis. Critically revising the work: Bingaman, Sharma, Rammo, Kondylis, Serletis. Reviewed submitted version of the work: Rammo, Sarmey, Kondylis, Serletis. Approved the final version of the work on behalf of all authors: Bingaman. Supervision: Serletis. Performed cadaveric dissections: Rammo.

## Supplemental Information

### Patient Informed Consent

The necessary patient informed consent was obtained in this study.
